# SARS-CoV-2 infections among asymptomatic individuals contributed to COVID-19 cases: A cross-sectional study among prospective air travelers from Ghana

**DOI:** 10.3389/fpubh.2022.1035763

**Published:** 2022-12-14

**Authors:** Kwasi A. Akowuah, Richard A. Akuffo, Anthony T. Boateng, Theodore W. Asigbee, Joseph H. K. Bonney, Helena Lamptey, Mildred A. Adusei-Poku, Evangeline Obodai, Ivy A. Asante, Samuel Adjei, James O. Aboagye, Susan Adu-Amankwah, Frederica D. Partey, George B. Kyei, William K. Ampofo, John K. Odoom, Evelyn Y. Bonney

**Affiliations:** ^1^Department of Virology, Noguchi Memorial Institute for Medical Research, University of Ghana, Accra, Ghana; ^2^Department of Epidemiology, Noguchi Memorial Institute for Medical Research, University of Ghana, Accra, Ghana; ^3^Medical and Scientific Research Centre, University of Ghana Medical Centre, Accra, Ghana; ^4^Department of Immunology, Noguchi Memorial Institute for Medical Research, University of Ghana, Accra, Ghana; ^5^Department of Medical Microbiology, School of Medicine, University of Ghana, Accra, Ghana; ^6^Department of Animal Experimentation, Noguchi Memorial Institute for Medical Research, University of Ghana, Accra, Ghana

**Keywords:** SARS-CoV-2, asymptomatic, air travelers, SARS-CoV-2 infection, COVID-19, Ghana

## Abstract

**Background:**

The spread of severe acute respiratory syndrome coronavirus 2 (SARS-CoV-2) by asymptomatic individuals has been reported since the early stages of the coronavirus disease 2019 (COVID-19) outbreak in various parts of the world. However, there are limited data regarding SARS-CoV-2 among asymptomatic individuals in Ghana. The aim of the study was to use test data of prospective travelers from Ghana as a proxy to estimate the contribution of asymptomatic cases to the spread of COVID-19.

**Methods:**

The study analyzed the SARS-CoV-2 PCR test data of clients whose purpose for testing was classified as “Travel” at the COVID-19 walk-in test center of the Noguchi Memorial Institute for Medical Research (NMIMR) from July 2020 to July 2021. These individuals requesting tests for travel generally had no clinical symptoms of COVID-19 at the time of testing. Data were processed and analyzed using Microsoft Excel office 16 and STATA version 16. Descriptive statistics were used to summarize data on test and demographic characteristics.

**Results:**

Out of 42,997 samples tested at the center within that period, 28,384 (66.0%) were classified as “Travel” tests. Of these, 1,900 (6.7%) tested positive for SARS-CoV-2. The majority (64.8%) of the “Travel” tests were requested by men. The men recorded a SARS-CoV-2 positivity of 6.9% compared to the 6.4% observed among women. Test requests for SARS-CoV-2 were received from all regions of Ghana, with a majority (83.3%) received from the Greater Accra Region. Although the Eastern region recorded the highest SARS-CoV-2 positivity rate of 8.35%, the Greater Accra region contributed 81% to the total number of SARS-CoV-2 positive cases detected within the period of study.

**Conclusion:**

Our study found substantial SARS-CoV-2 positivity among asymptomatic individuals who, without the requirement for a negative SARS-CoV-2 result for travel, would have no reason to test. These asymptomatic SARS-CoV-2-infected individuals could have traveled to other countries and unintentionally spread the virus. Our findings call for enhanced tracing and testing of asymptomatic contacts of individuals who tested positive for SARS-CoV-2.

## Background

Crucial to the transmissibility of COVID-19 is the high level of severe acute respiratory syndrome coronavirus 2 (SARS-CoV-2) shedding in the upper respiratory tract, even among presymptomatic patients, which differentiates it from SARS-CoV-1, where reproduction occurs primarily in the lower respiratory tract. Viral loads of SARS-CoV-1, which are associated with symptom onset, peak a median of 5 days later than that of SARS-CoV-2. This makes symptom-based detection of infection more effective in SARS-CoV-1 compared to SARS-CoV-2 (1).

The level of viral load among asymptomatic SARS-CoV-2-infected individuals has been shown to be similar to that of symptomatic individuals, suggesting that transmission potential among asymptomatically infected individuals may be significant (2, 3). This assumption led to the initial approach of controlling the disease by case isolation and quarantine measures that focused on symptomatic-confirmed cases only, rendering asymptomatic cases uncontrolled (4). However, there is strong evidence that asymptomatic individuals contribute significantly to the spread of SARS-CoV-2 (5–7).

Transmission of SARS-CoV-2 by infected, albeit asymptomatic individuals has been reported since the early stages of the outbreak resulting in what Gandhi et al. (8) referred to as the Achilles' heel of COVID-19 pandemic control (1). Thus, the role of asymptomatic individuals may not have been fully quantified but appears to be substantial enough in the spread of the COVID-19 disease particularly because such individuals do not usually present at healthcare or testing facilities for isolation or quarantine (1, 5–7). Asymptomatic SARS-CoV-2 infections are of great concern (9) because screening methods such as temperature checks tend to miss them; however, viral loads from their upper respiratory samples are usually comparable with those in symptomatic patients (10, 11). It is also documented that viral loads in throat samples, which are indicative of infectiousness, peak before or at the onset of symptoms (8), suggesting that asymptomatic carriers are a source of SARS-CoV-2 spread among households, workplaces, health centers, and in the community (12–15). Additionally, asymptomatic individuals may have a longer duration of virus shedding. Data from three Chinese hospitals, including 24 asymptomatic subjects, showed an average SARS-CoV-2 carrier period of 22 days. The time from exposure to eventual negativity shows that asymptomatically infected individuals are likely to carry the virus for a relatively long period (15).

Some studies have suggested that about 30–59% of SARS-CoV-2 infections are asymptomatic (9, 16–18) which creates tremendous infection control challenges (19, 20). Other studies found that asymptomatic individuals were the source of 69% of SARS-CoV-2 infections (21). Asymptomatic infections cannot be identified if they are not confirmed by reverse transcription polymerase chain reaction (RT-PCR) or another laboratory testing (22). However, in many countries, including Ghana, PCR testing to determine SARS-CoV-2 infection status has focused mainly on symptomatic patients (23).

The role of asymptomatic carriers in the spread of infectious diseases has been recognized in similar respiratory viral outbreaks before the SARS-CoV-2 pandemic (23). During the SARS-CoV outbreak in Singapore in 2003, 7.5% of serology-positive healthcare workers and 13% of cases in the general population were asymptomatic (24–26). Existing data suggest that SARS-CoV-2 can be highly contagious in individuals before symptom onset and in individuals who never develop symptoms (23, 27). With the emergence of the highly transmissible Omicron BA.2 strain of SARS-CoV-2, studies have shown higher rates of asymptomatic infections, particularly among the vaccinated ones (28, 29). Thus, focusing on testing and isolating symptomatic patients alone cannot control the SARS-CoV-2 pandemic (27).

International travel has played a major role in the spread of SARS-CoV-2 with evidence of SARS-CoV-2 importation documented in many studies, including one from Ghana (30–32). This led to travel restrictions and border control measures in many countries (33). While a study in Ghana reported 10.2% positivity among quarantined air travelers returning to Ghana, 21.5% of travelers tested positive in Canada during the mandatory quarantine period (30, 31).

In response to the COVID-19 pandemic, many countries including the United States of America recommended air travelers' test for SARS-CoV-2 before departure and upon arrival into the country (34). In line with this recommendation, the Noguchi Memorial Institute for Medical Research (NMIMR), Ghana's largest SARS-CoV-2 testing center, opened a walk-in testing center for the public, particularly air travelers, in July 2020. The core objective of the NMIMR walk-in center (NWC) was to test and generate a travel certificate for prospective air travelers from Ghana who needed to meet the travel requirements of the SARS-CoV-2 PCR test taken 48–72 h prior to boarding a flight.

The NWC also conducted tests on clients for other purposes aside from “TRAVEL.” Other test categories were classified as “SELF,” “MEDICAL,” and “WORK” if they were required for personal reasons, requested as part of medical appointments, or required for work-related reasons, other than travel. The tests categorized as “TRAVEL” were for individuals who reported no sign or symptom of COVID-19 but required the test to obtain a travel certificate, if negative. In addition to samples received through the NWC, the NMIMR also tested the samples received from clinically suspected or symptomatic individuals at various health facilities in Ghana.

This article reported data from a 13-month period (July 2020 to 2021) of SARS-CoV-2 tests conducted at the NWC to estimate the rate of SARS-CoV-2 positivity among asymptomatic COVID-19 cases using prospective air travelers from Ghana as a proxy. The intent is to infer the contribution of asymptomatic COVID-19 cases to the spread of SARS-CoV-2 infections in Ghana and propose strategies to enhance appropriate transmission control measures.

## Methods

### Study design

The study applied a cross-sectional analysis for SARS-CoV-2 PCR test data generated from the NWC from July 2020 to July 2021. The aim of the study was to use test data of prospective travelers from Ghana as a proxy for estimating asymptomatic COVID-19 cases. All clients visiting the center were required to provide demographic data including age/date of birth, sex, district of residence, reason for testing, nationality, and personal details such as telephone contact and passport numbers of travelers. These data were stored in the NMIMR's novel Coronavirus (nCoV) commercial test database. Only data from tests categorized as “TRAVEL” were analyzed for this study.

### Sample collection and testing

Both nasopharyngeal and oropharyngeal samples were taken from clients into a viral transport medium. The samples were transferred from the NWC, located behind the Advanced Research Laboratories (ARL) at NMIMR, to the Virology laboratories within the ARL for processing. Ribonucleic acid (RNA) was extracted from the samples according to the manufacturer's protocol. The list of RNA extraction kits used included QIAmp RNA (Qiagen, Germany), Viral Nucleic Acid (Geneaid, Taiwan), Nextractor NX-48 (Genolution, Korea), Viral RNA detection (DAAN Gene, China), Beaverhead Viral RNA/DNA (Beaver, China), Quick-RNA MiniPrep (Zymo, United States), and Veri-Q Prep M16 system (MiCo Biomed, Korea) based on availability. RNA was subjected to real-time RT-PCR (rRT-PCR) using primers and probes that target the SARS-CoV-2 nucleocapsid (N), the open-reading frame lab (ORF lab), and/or the envelope (E) genes. At least two of the targets were detected in the assay. The rRT-PCR result was interpreted as positive or negative according to the kits manufacturers' interpretation guidelines. The kits used were ModularDx (TIB MOLBIOL, Germany) and VeriQ PCR 316 nCoV-QS (MiCo Biomed, Korea). A cycle threshold (Ct) of <40 was considered positive for SARS-CoV-2.

### Statistical analysis

Data were processed and analyzed using Microsoft Excel office 16 and STATA version 16. Incomplete data, in the form of tests without sex, age, or region, were classified as “Unspecified” and filtered out. Tests that were not categorized but had passport details were categorized as “TRAVEL” as passport details were only required from prospective air travelers. However, tests that were not categorized and also had no passport details were categorized as “UNCATEGORIZED” and filtered out. Only tests categorized as “TRAVEL” were included in the analysis.

**Figure 1:**
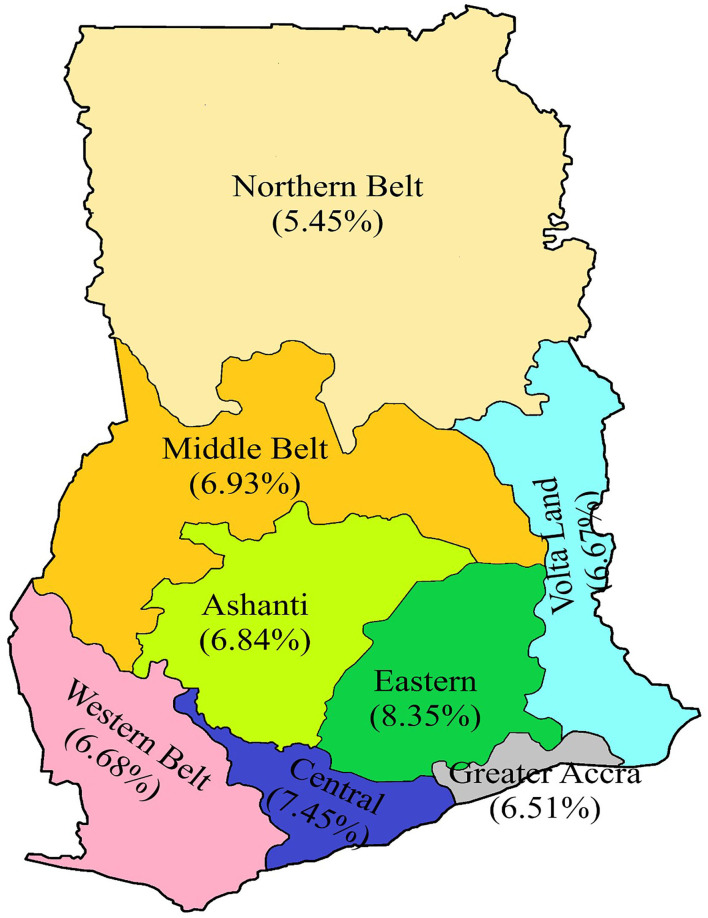
A revised map of Ghana showing the regional distribution of positivity rate. The Ahafo, Bono East, and Brong Ahafo regions are defined as the Middle belt; the Upper West, Upper East, North East, Savannah, and Northern regions were defined as the Northern belt. The Oti and Volta regions were defined as Volta Land, while the Western North and Western regions were defined as the Western belt. The remaining regions retained their administrative status and names.

Descriptive statistics were used to analyze the rate of positivity among prospective travelers and also among the various test categories. Inferential statistics (Pearson's chi-square test) was performed to determine the probability value of the data variables, and the association between SARS-CoV-2 positivity and other categorical variables at a significance level of 0.05. In computing the regional distribution of positivity for the purpose of this study, the Ahafo, Bono East, and Brong Ahafo regions were defined as the Middle belt. The Upper West, Upper East, North East, Savannah, and Northern regions were defined as the Northern Belt. The Oti and Volta regions were defined as Volta Land while the Western North and Western regions were defined as the Western Belt. The remaining regions retained their administrative status and names (Figure 1).

### Study variable

The study used PCR results for tests categorized as “TRAVEL” as the outcome variable, with positive referring to tests that were confirmed positive for SARS-CoV-2 by rRT-PCR. The study used variables collected for tests categorized as “TRAVEL” as the independent variables.

## Results

A total of 42,997 clients patronized the NWC from July 2020 to July 2021 and were tested for SARS-CoV-2. Of these, 28,384 (66%) were prospective air travelers. Testing for self-check was 20% and the remaining 14% tested for other reasons including “Medical” and “Work” (Table 1). Of the 28,384 prospective air travelers, 1,900 representing 6.69% tested positive for SARS-CoV-2 (Table 1).

**Table 1:** Test category distribution of SARS-CoV-2 PCR test at the NWC from July 2020 to July 2021.

**Test category** (*P* < 0.001***)	**Total tested** **(*n =* 42997)**	**Total positive** **(*n =* 4946)**	**Positivity rate (11.5%)**
Medical	444	148	33.33
Self	8927	2039	22.84
Travel	28384	1900	6.69
Work	2051	267	13.02
Uncategorized	3191	592	18.55

The male-to-female ratio of the travelers was 2:1. The majority (82.5%) of the clients were 20–59 years of age, with individuals aged 10–19 years showing the highest positivity rate of 7.27%. The Greater Accra region contributed 83.3% to the total number of clients tested for travel purposes (Table 2).

**Table 2:** Demographic and test characteristics of prospective air travelers who took SARS-CoV-2 PCR test at the NWC from July 2020 to July 2021.

	**Total tested**	**Total positive**	^*^Case proportion (%)	^**^Positivity rate (%)	***p-value***
Total (N)	28384	1900	1900		
**Sex (travel)**					
Male	18387	1263	66.47	6.87	
Female	9935	631	33.21	6.35	0.161
Unspecified	62	6	0.32	9.68	
**Age (travel)**					
0–9	481	30	1.58	6.24	
10–19	1238	90	4.74	7.27	
20–29	6346	452	23.79	7.12	0.251
30–39	7677	519	27.32	6.76	
40–49	5419	366	19.26	6.75	
50–59	3949	227	11.95	5.75	
60+	2466	158	8.32	6.41	
Unspecified	808	58	3.05	7.18	
**Region (travel)**					
Ashanti	892	61	3.21	6.84	
Central	1396	104	5.47	7.45	
Eastern	850	71	3.74	8.35	
Greater Accra	23639	1539	81.00	6.51	0.073
Middle belt	361	25	1.32	6.93	
Northern belt	110	6	0.32	5.45	
Volta land	90	6	0.32	6.67	
Western belt	374	25	1.32	6.68	
Unspecified	672	63	3.32	9.38	

* Case proportion is the number of positive cases for a category in relation to the total number of positive cases from travel.

** Positivity rate is the number of positive cases for a category in relation to the number of individuals tested in that category.

### Regional distribution of positive cases

The regional distribution of positivity rate among prospective travelers from Figure 1 shows the Eastern region recorded the highest positivity rate of 8.35%, while the Northern Belt recorded the lowest positivity rate of 5.45% (Figure 1). However, the Greater Accra region contributed the highest case proportion of 81% to the total positive cases among prospective travelers. On the other hand, both the Northern Belt and Volta Land contributed to the lowest proportion of 0.32% (Table 2).

### Monthly distribution of positive cases

**Figure 2:**
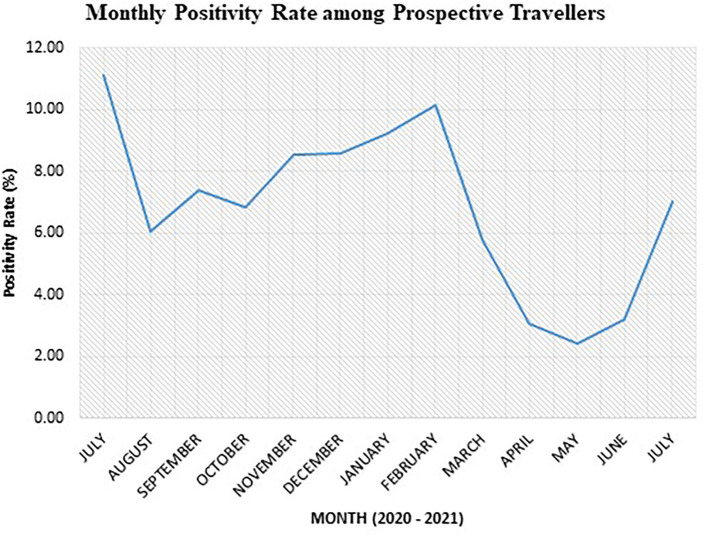
Monthly SARS-CoV-2 positivity rate among prospective air travelers from July 2020 to 2021.

Results from the monthly analysis of the “TRAVEL” test category showed the highest positivity rate among prospective travelers in July 2020 (11.11%), January 2021 (9.22%), and February 2021 (10.15%) (Figure 2). We recorded the highest positivity rate in July 2020, although the number of dlients tested was the lowest (*n* = 81) for the study period (Table 3). On the other hand, the lowest positivity rates among prospective travelers were recorded in the months of April, May, and June 2021.

**Table 3:** Monthly distribution of SARS-CoV-2 PCR test for prospective travelers at the NWC from July 2020 to July 2021.

**Year**	**2020**	**2020**	**2020**	**2020**	**2020**	**2020**	**2021**	**2021**	**2021**	**2021**	**2021**	**2021**	**2021**
Month	July	August	September	October	November	December	January	February	March	April	May	June	July
Total test	81	297	2261	2247	2070	2947	3632	2680	2512	2554	2478	2438	2187

After a high positivity rate of 11.11% in July 2020, the trend of positivity rate generally increased from November 2020 to February 2021. The lowest point of 2.42% was observed in May 2021. There was a sudden rise in the month of June 2021, followed by a sharp increase in July 2021.

It is worth noting that air travel from March 2020 to September 2020 was mostly restricted to chartered flights because Ghana's air border was closed. Hence, the total number of tests done for prospective travelers during that period was less compared to the period after the official opening (Table 3).

### Monthly sex-specific SARS-CoV-2 positivity rate

**Figure 3:**
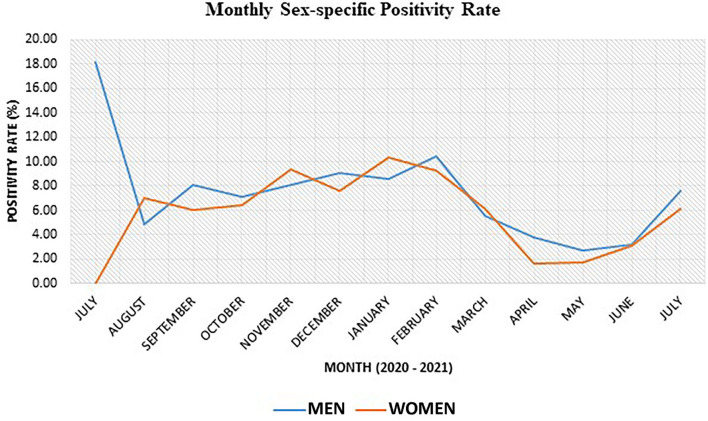
Sex-specific SARS-CoV-2 positivity rate for various months within the study period.

Generally, the SARS-CoV-2 positivity rate was 0.52% higher in men than in women (Figure 3). The monthly sex-specific positivity rate analysis shows that the positivity rate was higher in women for August 2020, November 2020, January 2021, and March 2021 (Figure 3).

## Discussion

This study estimated the rate of SARS-CoV-2 positivity among prospective travelers from Ghana who had their SARS-CoV-2 RT-PCR test at NWC from July 2020 to July 2021, which was used as a proxy for asymptomatic COVID-19 cases since these travelers had no known clinical symptoms for COVID-19 but only tested because of airline or destination requirements.

Our data showed the monthly positivity rate among prospective travelers reached a stable rate after October 2020 and peaked again in February 2021. This observation, to an extent, could be attributed to the campaign activities toward the presidential and parliamentary elections held in Ghana on 7 December 2020. There were mass political rallies toward the build-up to the election that peaked in November. Some of these gatherings may not have strictly adhered to the COVID-19 prevention protocols of social distancing, hand washing/sanitizing, and mask-wearing. This may account for the sharp rise in positivity observed in February 2021.

The detection of SARS-CoV-2 among asymptomatic individuals with the intention to travel is of public health concern since previous research has documented evidence of virus transmission by international travelers who were asymptomatic at the time of travel (2, 16, 21). Some of these patients tested PCR positive upon arrival at their destinations, while some tested positive during quarantine. Others tested positive only after their release from quarantine and yet each of these categories of individuals was responsible for some transmission events (31). Kiang et al. (35) studied the spread of SARS-CoV-2 in the absence of a PCR test before travel by simulating different scenarios and found that pretravel PCR tests reduced the transmission by 36% compared to when passengers did not take a test (35). These findings support our assertion that the asymptomatic positive cases found in our study were a potential source of transmission of the virus either during their flight (36, 37) or upon arrival at their destination (31). Although we did not trace the contacts of the positive asymptomatic cases in our study to check for transmission, we can confidently infer, based on previous studies mentioned earlier, that these individuals were a potential source of the spread of the virus if they had not tested as part of travel requirement.

As expected, more men than women took the SARS-CoV-2 test for the purpose of air travel because generally, men tend to undergo more foreign travel than women counterparts (38–40). Although both men and women travel to other countries, men tend to travel for work-related reasons, while women mostly travel for education or social reasons such as marriage (38, 41). The case proportion of men was twice than that of women probably because men tend to be more adventurous than women and are more likely to undertake high-risk behaviors such as not wearing face masks, attending parties, not keeping social distance, and generally not adhering to COVID-19 preventive protocols (42, 43). Our data also found that more than 70% of cases were identified among young and middle-aged individuals of 20–49 years, which is expected to be the most active period in life where people tend to associate more, travel more for education, business, or pleasure, and rationalize more risk (41–43).

The election-related activities of 2020 were followed by preparations for Christmas celebrations and end-of-year family gatherings. Perhaps, this could explain the marginal increase in the positivity rate in December.

One major characteristic of the Christmas season of 2020 was the inflow of individuals from various destinations overseas to the country, who probably introduced the Alpha variant, detected in Ghana in January 2021, which initiated the second wave of the COVID-19 pandemic. Despite a negative PCR test within a 72-h period required for air travel and the negative antigen test required upon arrival in Ghana, it is likely that some individuals infected with the virus might show SARS-CoV-2 negative at the time of the initial test as shown in other studies (31, 32). The introduction of the new variant to the population in Ghana may best explain the further increase in the positivity rate in January and February 2021. This period marked the country's second wave of SARS-CoV-2. The majority of these viruses were the Alpha strain first detected in the United Kingdom (44).

The abovementioned factors caused the government to tighten restrictions so as to help curb the upsurge of SARS-CoV-2 cases. This period led to the closure of all nightclubs, pubs, cinemas, and beaches that were operating in defiance of the law. These directives were captured in the presidential address (Update No. 22) delivered on Sunday, 17 January 2021, to the nation on updates to Ghana's enhanced response to the coronavirus pandemic (44). At that time, the Ghana Health Service was recording, on average, 200 new cases of SARS-CoV-2 infections daily (44).

The subsequent address to the nation, on 31 January 2021 (Update No. 23), reported average daily rates of infection stood at 700 cases (45). A ban was placed on funerals, weddings, concerts, theatrical performances, and parties. However, private burials, with not more than 25 people, were allowed with the enforcement of social distancing, handwashing or sanitizing, and mask-wearing protocols. All workplaces were directed to employ a shift system for workers, in addition to the use of virtual platforms for business or work, where possible (45).

With the restrictions in place, a decline in the positivity rate from March to May 2021 was observed, as shown in our data. The period of March 2021 also led to the rollout of the first phase of vaccination in Ghana (46). Some other nationals within Ghana and Ghanaians living abroad who visited Ghana had been fully or partially vaccinated. The introduction of vaccination had the potential of offering a false sense of security and may have also caused people to disregard preventive protocols. Thus, the vaccinated people got infected without severe symptoms or diseases and unknowingly transmitted the virus to the unvaccinated ones who then got severely ill. In June 2021, the rate of positivity started to rise. It was within this period that the Delta variant was detected in India and started spreading across the world. Ghana detected its first case of the imported Delta variants in June 2021 and later from the community (47). Therefore, June 2021 to July 2021 marked the third wave and was driven by the Delta strain of SARS-CoV-2 according to the release (Update 26) from The Presidency, Republic of Ghana (47).

The strength of our data lies in the fact that NMIMR is the largest SARS-CoV-2 testing facility in Ghana. The NWC conducted tests for individuals from various regions of the country making the data nationally representative for generalization.

Data on the SARS-CoV-2 positivity rate among prospective travelers from Ghana, as presented in this study, exclude tests conducted at other testing laboratories and only capture tests conducted at NMIMR.

Our data are limited by the fact that prospective travelers self-reported having or not having symptoms so there is the possibility of recall bias on their part. Also, researchers did not collect data on the purpose of travel; therefore, it is possible that some clients were traveling to seek medical care outside the country, which would have been categorized as “MEDICAL” instead of travel.

## Conclusion

Our data show that asymptomatic SARS-CoV-2 infections might have contributed substantially to the COVID-19 burden by fuelling the spread of the disease. Our findings indicate that without the requirement for a negative SARS-CoV-2 result as a prerequisite for travel, SARS-CoV-2-infected individuals could have traveled to other countries and increased the destination's case count by further fuelling the spread of the virus.

Enhanced tracing and regular testing of both symptomatic and asymptomatic contacts of positive cases would promote early detection and avert the spread of the virus in Ghana.

## Data availability statement

The original contributions presented in the study are included in the article/supplementary material, further inquiries can be directed to the corresponding author.

## Ethics statement

The study was done as part of emergency pandemic response accordance to the Ghana Health Service directives for regarding international travel and so did not require a special IRB clearance or written informed consent. This study reports results from prospective travelers who took a PCR test at our center under this provision.

## Author contributions

EB, JB, IA, RA, and WA conceptualized the study. KA, RA EB, JO, GK, and WA designed the study. TA, KA, AB, and MA-P performed the testing. JO, WA, IA, SA, SA-A, HL, MA-P, EO, JA, and FP supervised the testing. KA, AB, TA, GK, and RA analyzed the data. KA, AB, TA, and EB wrote the draft. All authors reviewed and improved the draft and approved the final version for publication.
